# Teleoperation of High-Speed Robot Hand with High-Speed Finger Position Recognition and High-Accuracy Grasp Type Estimation

**DOI:** 10.3390/s22103777

**Published:** 2022-05-16

**Authors:** Yuji Yamakawa, Koki Yoshida

**Affiliations:** 1 Interfaculty Initiative in Information Studies, The University of Tokyo, Tokyo 153-8505, Japan; 2 School of Engineering, The University of Tokyo, Tokyo 153-8505, Japan; k.yoshida.890@gmail.com

**Keywords:** teleoperation, high-speed image processing, machine learning, finger position recognition, grasp type estimation

## Abstract

This paper focuses on the teleoperation of a robot hand on the basis of finger position recognition and grasp type estimation. For the finger position recognition, we propose a new method that fuses machine learning and high-speed image-processing techniques. Furthermore, we propose a grasp type estimation method according to the results of the finger position recognition by using decision tree. We developed a teleoperation system with high speed and high responsiveness according to the results of the finger position recognition and grasp type estimation. By using the proposed method and system, we achieved teleoperation of a high-speed robot hand. In particular, we achieved teleoperated robot hand control beyond the speed of human hand motion.

## 1. Introduction

Technology for realizing remote systems such as teleoperation, telerobotics, telexistence, etc., has been an important issue [1,2,3], and much research has been actively carried out. In the recent situation, in particular with the effects of COVID-19, remote work (tele-work) by office workers has become commonplace. In the future, teleoperation using robot technology will be applied to industrial fields, and object handling and manipulation using remote systems are considered to be essential and critical tasks. In order to achieve this, we consider that telerobotics technology based on sensing human hand motion and controlling a robot hand will be essential. Thus, this research focuses on the teleoperation of a robot hand on the basis of visual information about human hand motion. The reason why we use visual information is that it is troublesome for users to have to put on contact devices [4,5,6,7] before operating the system, and non-contact-type systems are considered to be more suitable for users.

Here, we describe related work in the fields of teleoperation and telerobotics based on visual information. Interfaces based on non-contact sensing generally recognize human hand gestures and control a slave robot based on these gestures [8,9]. In the related work in the field of humanoid robotics, a low-cost teleoperated control system for a humanoid robot has been developed [10]. In wearable robotics, semantic segmentation has been performed by using Convolutional Neural Networks (CNNs) [11]. Such interfaces are intuitive for users and do not involve the restrictions involved with contact-type input devices. Some devices for recognizing human hand gestures have been developed, and some systems have been also constructed [12,13]. Lien et al. proposed a high-speed (10,000 Hz) gesture recognition method based on the position change of the hand and fingers by radar [14]. This method can recognize the rough hand motion, but not its detail. Zhang et al. performed human hand and finger tracking using a machine learning technique based on RGB images, but the operating speed was limited to 30 fps [15]. Tamaki et al. created a database consisting of finger joint angles obtained by using a data glove, hand contour information, and nail positions obtained from images, and they also proposed a method of estimating hand and finger positions by searching the database at 100 fps [16].

Premeratne discussed some techniques for hand gesture recognition for Human–Computer Interaction (HCI) [17]. Furthermore, Ankit described recent activities on hand gesture recognition for robot hand control [18]. Hoshino et al. [19] and Griffin et al. [20] proposed methods of mapping between human hand and robot hand motions. On the other hand, Meeker et al. [21] created a mapping algorithm experimentally. Sean et al. developed a system that can operate a robot arm according to human intention [22]. Niwa et al. proposed “Tsumori” control, which can achieve a unique robot operation for an operator based on learning the correspondence of a human operation and robot motion [23]. Fallahinia and Mascaro proposed a method of estimating hand grasping power based on the nail color [24].

Summarizing the above, we can conclude that the disadvantages of the previous approaches are as follows:

1. Low speed: The sampling rate is course, and the gain of the robot controller becomes small, resulting in low responsiveness.
2. Low responsiveness: The latency from the human motion to the robot motion is long, making it difficult to remotely operate the robot. Furthermore, the system cannot respond to rapid and random human motion.

Regarding the low speed and low responsiveness, Anvari et al. [25] and Lum et al. [26] discussed the system latency in surgical robotics, and they claimed that the latency affects the task completion and performance. Thus, it is strongly desirable for teleoperation systems to have as low a system latency as possible.

To overcome these disadvantages, we also developed a high-speed telemanipulation robot hand system consisting of a stereo high-speed vision system, a high-speed robot hand, and a real-time controller [27,28]. In the stereo high-speed vision system, which is composed of two high-speed cameras and an image-processing PC, the 3D positions of the fingertips of a human subject were calculated by a triangulation method. Then, mapping between the human hand and the robot hand was performed. Finally, robot hand motion was generated to duplicate the human hand motion. With this high-speed system, we achieved a system latency so low that a human being cannot recognize the latency from the human hand motion to the robot hand motion [29,30].

In the present research, we aim to achieve even lower latency so that an intelligent system with vision cannot recognize the latency. Realizing such an extremely low-latency teleoperated system will contribute to solutions for overcoming latency issues in cases where the latency of telemanipulated systems may occur in more distant places. In addition, this technology will enable high-level image processing using the remaining processing time. In this paper, we propose a new method that fuses machine learning and high-speed image-processing techniques to obtain visual information about human hand motion. In general, the speed of machine learning methods is considered to be very low, and therefore, we consider that it is not suitable to adapt machine learning methods for real-time and real-world interactions between a human and a robot. By using our proposed method, we can overcome the issue with the low speed of the machine learning processing. Concretely speaking, the low-speed characteristics of machine learning can be improved by using high-speed image processing and interpolating the results of the machine learning with the results of the high-speed image processing. Although the finger position is estimated by machine learning using a CNN and high-speed image-processing technologies in this research, the integration of machine learning and high-speed image-processing technologies can be considered to be applicable to other target tracking tasks. Thus, our proposed method with high speed and high intelligence possesses the generality of the target-tracking method.

In addition, since our proposed method does not require three-dimensional measurement and camera calibration is also not needed, it is easy to set up the system. Moreover, motion mapping from the human hand motion to the robot hand motion is not performed in our proposed method. Therefore, kinematic models of the human hand and robot hand are not needed either. As a result, it is considered to be easy to implement our developed teleoperation system in actual situations.

The contributions of this paper are the following:

1. Integration of a machine learning technique and high-speed image processing;
2. High-speed finger tracking using the integrated image processing;
3. High-accuracy grasp type estimation;
4. Real-time teleoperation of a high-speed robot hand system;
5. Evaluation of the developed teleoperation system.

Furthermore, Table 1 shows the positioning of this research. The characteristics of our proposed method are “non-contact”, “intention extraction”, and “high-speed”.

The rest of this paper is organized as follows: Section 2 describes an experimental system for teleoperation. Section 3 explains a new method for achieving grasp type estimation based on high-speed finger position recognition. Section 4 shows evaluations of the proposed method and the experimental results of teleoperation. Section 5 concludes with a summary of this research and future work.

## 2. Experimental System

**Figure 1:**
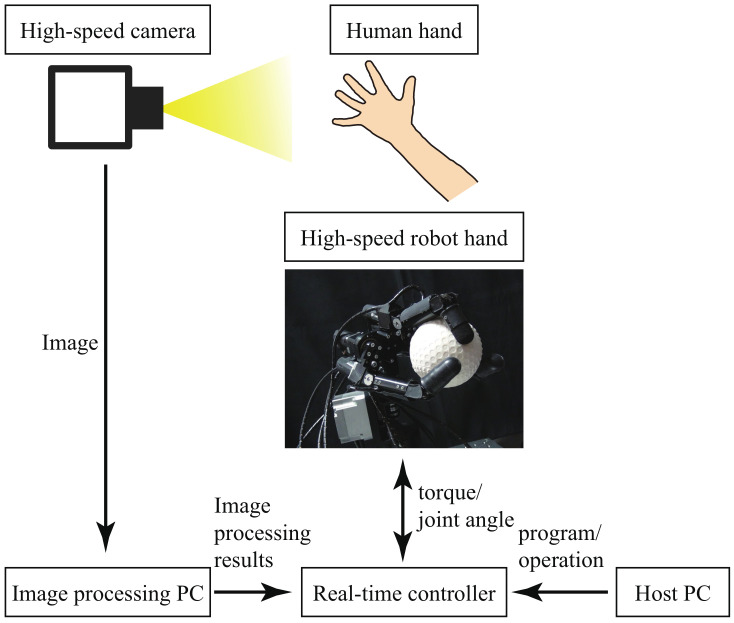
Structure of the experimental system.

This section explains our experimental system for the teleoperation of a high-speed robot hand based on finger position recognition and grasp type estimation. The experimental system, as shown in Figure 1, consists of a high-speed vision system (Section 2.1), a high-speed robot hand (Section 2.2), and a real-time controller (Section 2.3). All of the components were placed in the same experimental environment.

### 2.1. High-Speed Vision System

This subsection explains the high-speed vision system, consisting of a high-speed camera and an image-processing PC. As the high-speed camera, we used a commercial product (MQ013MG-ON) manufactured by Ximea. The full image size was 1280 pixels (width) × 1024 pixels (height), and the frame rate at the full image size was 210 frames per second (fps). In this research, since we decreased the image size, we increased the frame rate from 210 fps to 1000 fps. The reason why we set the frame rate at 1000 fps is that the servo control systems for the robot and machine system were both operated at 1000 Hz. In general, the raw image acquired by the high-speed camera was dark because of the significantly short exposure time. Therefore, we used an LED light to obtain brighter raw images from the high-speed camera.

The raw image data acquired by this high-speed camera were transferred to the image-processing PC. The image-processing PC ran high-speed image processing to track the finger position and to estimate the grasp type. The details of the image processing are explained in Section 3. The results of the image processing were sent to a real-time controller, described in Section 2.3. By performing real-time, high-speed (1000 Hz) image processing, we could control the high-speed robot hand described in Section 2.2 at 1 kHz. The sampling frequency of 1 kHz was the same as the sampling frequency of the servo-motor control.

The specifications of the image-processing PC are as follows: Dell XPS 13 9360, CPU: Intel® Core (™) i7-8550U @1.80 GHz, RAM: 16.0 GB, OS: Windows 10 Pro, 64 bit.

### 2.2. High-Speed Robot Hand

This subsection describes the high-speed robot hand, which was composed of three fingers [31]. A photograph of the high-speed robot hand is shown in the center of Figure 1. The number of degrees of freedom (DoF) of the robot hand was 10; the middle finger had 2 DoF, the left and right fingers 3 DoF, and the wrist 2 DoF. The joints of the robot hand could be closed by 180 degrees in 0.1 s, which is fast motion performance beyond that possible by a human. Each joint angle of the robot hand was controlled using a Proportional and Derivative (PD) control law, given by

(1) $$\tau =k_{p}\left(\theta_{d}-\theta \right)+k_{d}\left({\dot{\theta }}_{d}-\dot{\theta }\right),$$

where τ is the torque input as the control input for the high-speed robot hand control, $\theta_{d}$ and θ are the reference and actual joint angles of the finger of the robot hand, and $k_{p}$ and $k_{d}$ are the proportional and derivative gains of the PD controller.

### 2.3. Real-Time Controller

As the real-time controller, we used a commercial product manufactured by dSPACE. The real-time controller had a counter board (reading encoder attached to the motors of the robot hand), digital-to-analog (DA) output, and two Ethernet connections (one was connected to the host PC and the other to the image-processing PC). We operated the real-time controller through the host PC, and we also implemented the program of the proposed method in the host PC.

The real-time controller received the results of image processing via Ethernet communication. Then, the real-time controller generated a control signal for the robot hand to appropriately control the robot hand according to the results of the image processing and output the control signal to the robot hand.

## 3. Grasp Type Estimation Based on High-Speed Finger Position Recognition

This section explains a new method for estimating grasp type, such as power grasp or precision grasp, based on high-speed finger position recognition using machine learning and high-speed image processing, and our proposed method can be mainly divided into two components: high-speed finger position recognition described in Section 3.1 and grasp type estimation described in Section 3.2.

**Figure 2:**
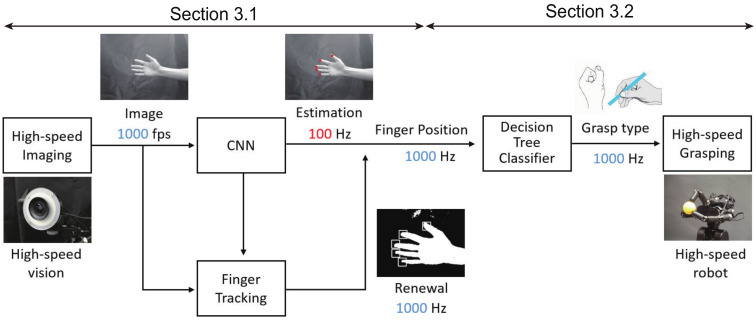
Overall flow of the proposed teleoperation method.

Figure 2 shows the overall flow of the proposed teleoperation method, detailed below:

1. Acquisition of the image by the high-speed camera:
  First, images can be captured by the high-speed camera at 1000 fps.
2. Estimation of finger position by CNN and finger tracking by high-speed image processing:
  The CNN and finger tracking are executed on the images. The calculation process of the CNN is run at 100 Hz, and finger tracking is run at 1000 Hz; the results of the CNN are interpolated by using the results of finger tracking. As a result, the finger positions are recognized at 1000 Hz.
3. Estimation of grasp type by decision tree classifier:
  Based on the finger positions, grasp type estimation is performed by using a decision tree classifier.
4. Grasping motion of the high-speed robot hand:
  According to the estimated grasp type, the high-speed robot hand is controlled to grasp the object.

### 3.1. High-Speed Finger Position Recognition with CNN

This subsection explains the method for high-speed finger position recognition. By recognizing the finger positions at high speed (for instance, 1000 fps), we can reduce the latency from the human hand motion to the robot hand motion and estimate the grasp type with high accuracy. Conventional image-processing methods are too slow (around 30 fps) for actual application. This research can solve the speed issue with the image processing conventionally used.

The proposed method was implemented by using machine learning and high-speed image-processing technologies. As the machine learning method, we used a Convolutional Neural Network (CNN). As the high-speed image processing method, we used tracking of a Region Of Interest (ROI) and image processing of the ROI. Here, the ROI was extremely small for the full image size and was set at the position of the result of the CNN, namely roughly at the position of the fingers.

#### 3.1.1. Estimation of Finger Position by CNN

By using the CNN, we estimated the positions of six points (five fingertips and the center position of the palm) in the 2D image captured by the high-speed camera. The advantages of using the CNN to recognize hand positions include robustness against finger-to-finger occlusion and robustness against background effects.

The model architecture of the CNN is as follows:

- Input: an array of 128 × 128 × 1;
- Output: 12 values;
- Alternating layers: six Convolution layers and six Max Pooling layers;
- Dropout layer placed before the output layer;
- The filter size of the Convolution layers was 3 × 3, the number of filters 32, and the stride 1;
- The pool size of Max Pooling was 2 × 2.

This architecture was created by referring to a model [32] used for image classification and modifying it according to handling multiple-output regression problems.

In addition, the value of Dropout was set at 0.1, and the activation function and parameter optimization were Relu and RMSprop [33], respectively. The loss was calculated using the Mean-Squared Error (MSE). Adding the Dropout layer was expected to suppress overlearning, and reducing the number of layers was expected to be effective in suppressing overlearning and reducing inference and learning times [34].

**Figure 3:**
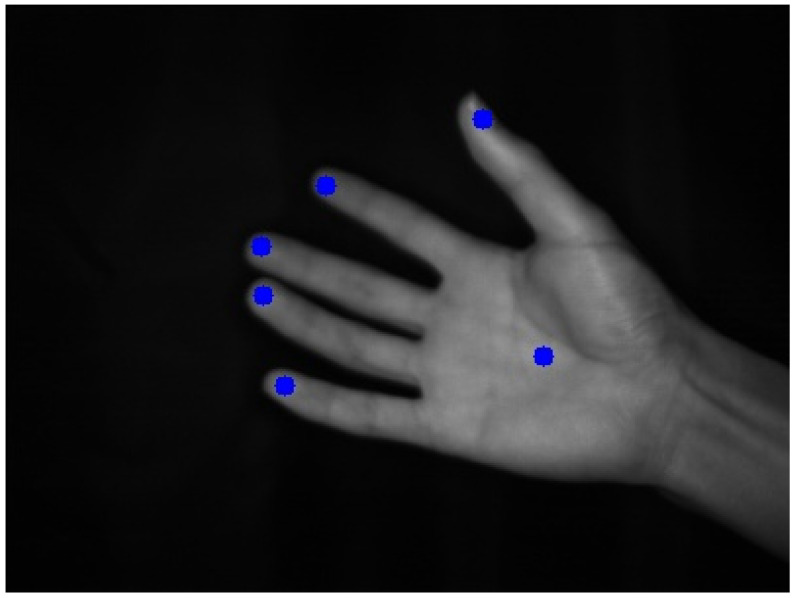
Annotation result of fingertip positions.

Figure 3 shows an example of annotation on a hand image, where the annotated positions indicating the center points of the fingertips and palms of the five fingers are shown as blue dots.

#### 3.1.2. Finger Tracking by High-Speed Image Processing

**Figure 4:**
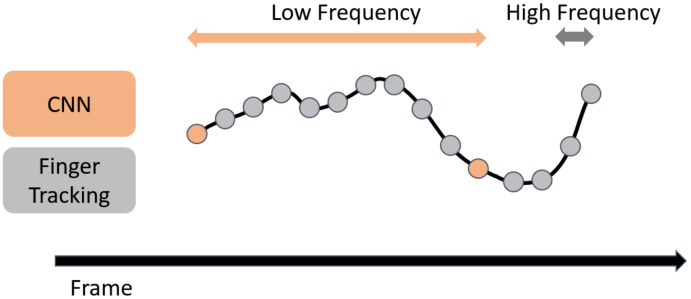
Concept of fusing the CNN and finger tracking.

Since the estimation by the CNN is much slower than imaging by the high-speed camera and there are many frames where the hand position cannot be recognized during the estimation by the CNN, the CNN processing becomes the rate-limiting step of the system. While compensating for the frames where the CNN estimation is not performed, we achieved real-time acquisition of the hand position. Figure 4 [35] shows a schematic of the method that combines CNN estimation performed at low frequency with high-frequency hand tracking to obtain the hand position. In Figure 4, the orange dots indicate the execution of the CNN, and the gray dots indicate the execution of hand tracking. By performing hand tracking in frames where the CNN is not performed, the hand position can be obtained at high frequency.

Next, we explain the requirements that the hand-tracking method should satisfy. First, in order to perform real-time hand position recognition, information obtained in frames after the frame to be tracked cannot be used. For example, if we perform linear interpolation of two CNN results to interpolate the hand position in the frame between the CNN steps, we need to wait for the second CNN to be executed, which impairs the real-time performance of the system. Therefore, it is necessary to track the hand based on the information obtained from the frame to be processed and the earlier frames.

In addition, it is desirable to obtain the data by measurement rather than by prediction. This is because it is not always possible to accurately recognize high-speed and minute hand movements if the current hand position is inferred from the trend of past hand positions. By calculating the current hand position from the information from the current frame, instead of predicting based on the information from the past frames, we can achieve more accurate recognition of sudden hand movements.

In this study, we propose a real-time, measurement-based recognition method for hand positions in frames where the CNN is not performed. This method involves hand tracking using frame-to-frame differences of fingertip center-of-gravity positions. The proposed hand-tracking method calculates the hand position in the corresponding frame by using three data sets: the hand position from the past CNN estimation results, the image of the frame in which the CNN was executed, and the image of the corresponding frame. As the amount of hand movement between frames, we calculated the difference in hand positions from the two images and added it to the hand position estimated by the CNN to treat it as the hand position in the corresponding frame.

**Figure 5:**
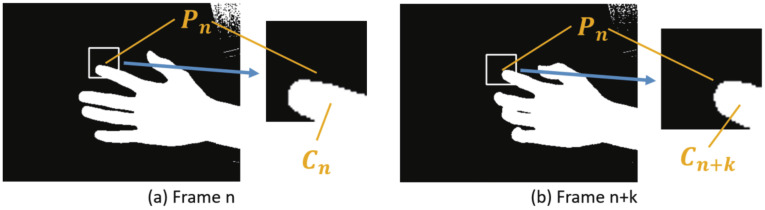
An example of finger tracking: (**a**,**b**) show images at the *n*-th and (n+k)-th frames, respectively.

The following is the specific method of calculating the hand position when the most recent frame in which the CNN is executed is *n*, the frame in which the tracking process is performed is n+k, and the frame in which the CNN is executed again is n+T:

1. *n*-th frame: CNN
  In the *n*-th frame, let an estimated fingertip position obtained by the CNN be $P_{n}$. Using Equation (2) below, the image is binarized, the ROI with the center position $P_{n}$ is extracted, and the center of the fingertip in the ROI is assumed to be $C_{n}$ (Figure 5a). In the image binarization, the original image and the binarized image are src(i,j) and f(i.j), respectively. Furthermore, the threshold of the image binarization is set at thre.
  (2) $$f\left(i,j\right)=\left\{\begin{array}{cc} 0 & \mathrm{if}\;\mathrm{src}(i,j)<thre\\ 1 & \mathrm{otherwise} \end{array}\right.$$
  The image moment is represented by $m_{pq}$ (Equation (3)), and the center position ($C_{n}$) of the fingertip in the ROI is $\left(m_{10}/m_{00},m_{01}/m_{00}\right)$:
  (3) $$m_{pq}=\sum_{i}\sum_{j}i^{p}j^{q}f\left(i,j\right)$$
  The value of $P_{n}$ is substituted for the fingertip position $Q_{n}$ in the *n*-th frame.
  (4) $$Q_{n}=P_{n}$$
2. (n+k)-th frame: Finger tracking
  After binarizing the image in the (n+k)-th frame (0<k<T), the ROI with the center position $P_{n}$ is extracted, and the center of the fingertip in the ROI is assumed to be $C_{n+k}$ (Figure 5b). At that time, let the finger position in the (n+k)-th frame be $Q_{n+k}$, calculated by the following equation:
  (5) $$Q_{n+k}=P_{n}+C_{n+k}-C_{n}$$

If the hand-tracking module receives a new CNN estimation result from the CNN module every *T* frames, where *T* is a predefined constant, and updates the result for processing, the hand-tracking process will have to wait for every frame that exceeds *T* for inference by the CNN. When the number of frames required for inference by the CNN exceeds *T*, the hand-tracking process needs to wait. This increases the latency of hand tracking, since the inference time of the CNN may vary in actual execution and the inference result may not be sent by the CNN module even after *T* frames have passed. On the other hand, if the CNN results are updated in frames received from the CNN module instead of in frames at regular intervals, the latency is reduced because the last received CNN result is used even if the CNN result is delayed. The latency is reduced because the last received CNN result is used even if the transmission of the CNN result is delayed.

Based on the above, we devised two different methods for hand tracking with and without a waiting time for the CNN estimation results: a low-latency mode with a variable *T* value and a high-accuracy mode with a constant *T* value. The low-latency mode is effective for applications where low latency is more important than accuracy, such as anticipating human actions. On the other hand, the high-accuracy mode is suitable for applications where accurate acquisition of hand positions is more important than low latency, such as precise mechanical operations. In this research, we adopted the low-latency mode to track the human hand motion, because we aimed at the development of a teleoperation system with high speed and low latency. An overview of the algorithm for the low-latency mode is shown in Algorithm 1. The algorithm for the high-accuracy mode is shown in Algorithm A1 in Appendix A.
**Algorithm 1** Finger tracking with low latency1:resultCNN {CNN result to receive}2:resultFT {Finger tracking result to send}3:**while** True **do**4:   **if** resultCNN is received **then**5:       calculate $C_{n}$6:       resultFT←resultCNN7:   **else**8:       calculate $C_{n+k}$9:       $resultFT\leftarrow resultCNN+C_{n+k}-C_{n}$10:   **end if**11:   send resultFT12:**end while**

The characteristics of the two modes are summarized below:
Algorithm 1. Low-latency mode:
As the result of the CNN, which is used for finger tracking, the latest result is utilized. The advantage is that the latency is reduced because no time is required to wait for the CNN results. The disadvantage is that if the CNN processing is delayed, the tracking process will be based on the CNN results for distant frames, which will reduce the accuracy.
Algorithm A1. High-accuracy mode:
By fixing the interval *T* of the number of frames at which the CNN is executed, the process of updating the estimate by CNN is performed at fixed intervals. The advantage is that hand tracking is based on frequently acquired CNNs, which improves accuracy. The disadvantage is that when the CNN processing is delayed, the latency increases because there is a waiting time for updating the CNN results before the tracking process starts.

### 3.2. Grasp Type Estimation

This subsection explains the method for estimating the grasp type on the basis of the results of the high-speed finger tracking. Furthermore, we explain the robot hand motion according to the estimated grasp type.

#### 3.2.1. Estimation of Grasp Type by Decision Tree

**Figure 6:**
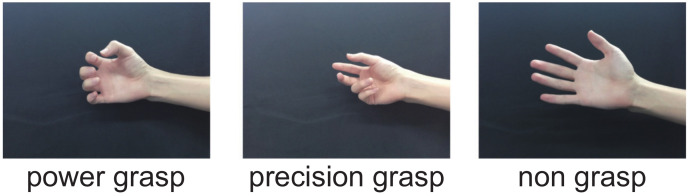
Differences among power grasp (**left**), precision grasp (**middle**), and non-grasp (**right**).

We also used machine learning technology to estimate the grasp type on the basis of the finger position and the center position of the palm, which are estimated by the CNN and hand tracking at high speed. As representative grasp types to be estimated, we considered two grasp types: (1) a power grasp using the palm of the hand and (2) a precision grasp using only the fingertips. As a result, we categorized the grasps to be estimated into three types: “power grasp”, “precision grasp”, and “non-grasp”, as shown in Figure 6. In the “power grasp”, all four fingers, and not the thumb, move in the same way and tend to face the thumb, whereas in the “precision grasp”, the positions of the thumb and index finger tend to separate from those of the little finger and ring finger. The “non-grasp” state corresponds to the extended state of the fingers.

To accurately estimate the grasp type, we used decision trees (decision tree classifier) as the machine learning method. Decision trees have the features of fast classification and readability of the estimation criteria. In particular, the ease of interpretation of the estimation criteria and the possibility of creating algorithms with adjustments are reasons for using decision trees as a classification method.

**Figure 7:**
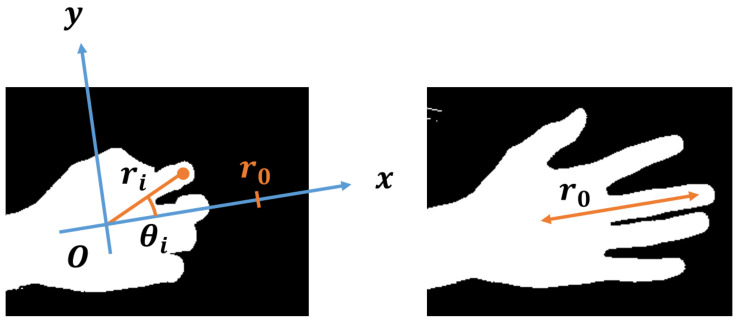
Direction of middle finger and angle between each finger and middle finger.

   Preprocessing of hand position data:

In order to improve the accuracy of the decision tree, we preprocessed the input data, namely the hand positions (Figure 7). In the preprocessing, we first calculate the distance between the middle finger and the palm of the hand in the frame with the fingers extended as a hand size reference to calibrate the hand size. When the coordinates of the middle finger and the palm of the hand in the image of the frame to be calibrated are $\left(x_{m0},y_{m0}\right)$ and $\left(x_{p0},y_{p0}\right)$, respectively, the distance between the two points can be given by

(6) $$r_{0}=\sqrt{{\left(x_{m0}-x_{p0}\right)}^{2}+{\left(y_{m0}-y_{p0}\right)}^{2}}.$$

Next, we calculate the position of the hand, (r,θ), in the polar coordinate system with the palm of the hand serving as the origin and the direction of extension of the middle finger serving as the *x*-axis, based on the position of the hand represented by the coordinates in the image. When the coordinates of the middle finger and the palm in the image are $\left(x_{m},y_{m}\right)$ and $\left(x_{p},y_{p}\right)$, respectively, the declination angle of the polar coordinate of the middle finger, $\theta_{m}$, is expressed by the following formula:

(7) $$\theta_{m}=\arctan\left(\frac{y_{m}-y_{p}}{x_{m}-x_{p}}\right).$$

Furthermore, the polar coordinate $\left(r_{i},\theta_{i}\right)$ corresponding to the coordinate $\left(x_{i},y_{i}\right)$ of finger *i* in the image is expressed by the following equation with the direction of the middle fingertip serving as the positive direction of the *x*-axis. To calibrate the size of the hand, we divide $r_{i}$ by the hand size reference $r_{0}$.

(8) $$\begin{array}{ccc} r_{i} & = & \frac{1}{r_{0}}\sqrt{{\left(x_{i}-x_{p}\right)}^{2}+{\left(y_{i}-y_{p}\right)}^{2}} \end{array}$$

(9) $$\begin{array}{ccc} \theta_{i} & = & \arctan\left(\frac{y_{i}-y_{p}}{x_{i}-x_{p}}\right)-\theta_{m} \end{array}$$

The vector r containing the distance ($r_{i}$) of each finger and the vector Θ containing the declination angle ($\theta_{i}$) obtained in this way for each frame are used as inputs to the decision tree. By using the relative positions of the fingers with respect to the palm in polar coordinates as inputs, the data to be focused on are clarified, and by dividing the data by the size of the hand with the fingers extended, the characteristics of the hand morphology can be extracted while suppressing the effects of differences in hand size between individuals and differences in the distance between fingers and camera lens for each execution.

#### 3.2.2. Grasping Motion of High-Speed Robot Hand

The object is grasped by the high-speed robot hand according to the result of the high-speed grasp type estimation described above.

The middle finger of the robot hand has two joints, that is the root and tip links, and the left and right fingers also have three joints, the root, tip, and rotation around the palm. The root and tip links operate in the vertical direction to bend and stretch the fingers, allowing them to wrap around objects. The rotation joint around the palm moves horizontally and can change its angle to face the middle finger, which enables stable grasping. The wrist part of the robot hand has two joints, that is flexion/extension and rotation joints, which enables the finger to move closer to the object to be grasped.

Since the time required to rotate the finger joint 180 deg is 0.1 s, it takes approximately 50 ms to close the finger from the open position to 90 deg for grasping. From our research using a high-speed control system, the latency from the image input to the torque input of the robot hand is about 3 ms [36]. If the value of the estimated grasping configuration oscillates, the target angle is frequently changed, and the robot hand becomes unstable, so the grasping operation is started when the same grasp type is received continuously for a certain number of frames.

## 4. Experiments and Evaluations

**Figure 8:**
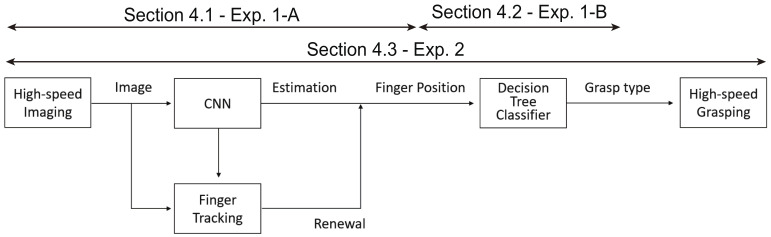
Overview of experiments and evaluations.

This section explains the experiments, experimental results, and evaluations for finger position recognition (Section 4.1—Exp. 1-A), grasp type estimation (Section 4.2—Exp. 1-B), and teleoperated grasping by a robot hand (Section 4.3—Exp. 2), respectively. Figure 8 shows an overview of the experiments and evaluations for each part.

### 4.1. Finger Position Recognition

This subsection explains the experiment and evaluation for finger position recognition based on the proposed method with high-speed image processing and the CNN.

#### 4.1.1. Preparation for Experiment

We trained the CNN model described in Section 3, which estimated hand positions from images. To increase the amount of data for training, we performed data augmentation by random scaling (0.7∼1.0) and rotation (−60∼60 deg.) operations. As a result, we could obtain 9000 images from 1000 images by data augmentation. The training process was performed for 200 epochs, with 70% of the prepared data used as the training data and 30% used as the validation data. The slope of the loss function calculated from the Mean-Squared Error (MSE) became lower around epoch 30. When we calculated the Mean Absolute Error (MAE) of the estimation results for the validation data, the MAE was less than 10 pixels. The width of the fingers in the image was about 15 pixels, which means that the hand position estimation was accurate enough for the hand-tracking process. As a result of 5 trials of 100 consecutive inferences, the mean and standard deviation of the inference times were 7.03 ms and 1.46 ms, respectively. Furthermore, the longest was 24.6 ms, and the shortest was 3.75 ms. Thus, an average of seven hand-tracking runs was taken for each update of the CNN results for 1000 fps of image acquisition by the high-speed camera.

#### 4.1.2. Experiment—1-A

The exposure time of the high-speed camera was set to 0.5 ms, the image size to 400 pixels wide and 300 pixels high, the square ROI for the hand-tracking process to 40 pixels by 40 pixels, and the threshold for the binarization process to thre=20. In such a situation, we captured the hand opening and closing in 1000 frames during 1 s and applied the proposed hand tracking for hand position recognition. The images captured in the experiment were stored, the CNN was run offline on all images, and the results were used as reference data for the comparison method.

#### 4.1.3. Results

The output speed of the hand position was 1000 Hz, which was the same speed as the imaging. The latency between the end of imaging and the output of the hand position was also 1 ms.

**Figure 9:**
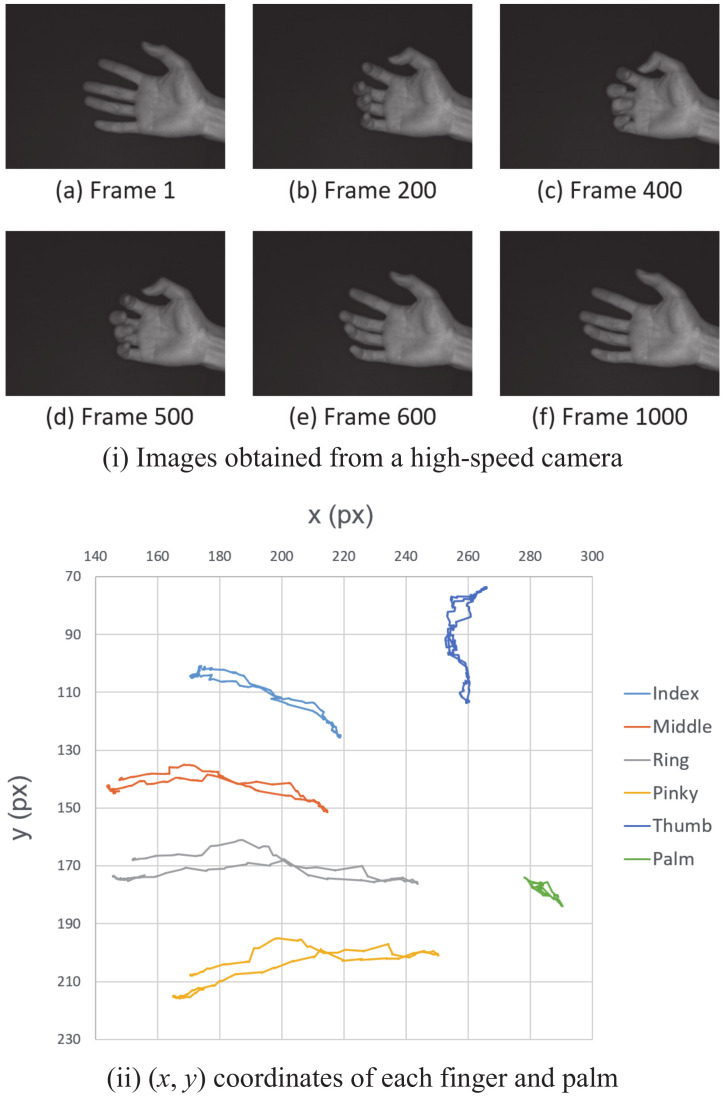
Result of finger tracking: (**i**) images obtained from the high-speed camera and (**ii**) (*x*, *y*) coordinates of each finger and the palm.

Hand images are shown in Figure 9i: starting with the finger extended (Figure 9(i-a)), bending the finger (Figure 9(i-b)), folding it back around Frame 400 (Figure 9(i-c)), and stretched again (Figure 9(i-d)–(i-f)). Figure 9ii is a graph of the hand positions estimated over 1000 frames by the proposed method. The image coordinates of the five fingers and the palm center point are represented as light blue for the index finger, orange for the middle finger, gray for the ring finger, yellow for the pinky finger, dark blue for the thumb, and green for the palm.

The errors in the proposed method and the errors in the comparison method are shown in Table 2. The average error of the five fingers in the proposed method was 1.06 pixels. Furthermore, the error in the comparison method was 1.27 pixels, which is 17% bigger than that in the proposed method. For all fingers, the error in the proposed method was smaller than that of the comparison method. Note that 1 pixel in the hand image corresponds to approximately 0.7 mm in the real world.

#### 4.1.4. Discussion

First, we consider the execution speed of hand position recognition. From the experimental results, the output speed of the hand position was 1000Hz, which is the same as that of the image capturing, indicating that the hand position recognition is fast enough. In addition, the latency from the end of imaging to the output of the hand position was 1ms, indicating that the total execution time of the inter-process data sharing and hand-tracking process itself was 1ms. Thus, the effectiveness of the proposed method described in Section 3 was shown.

Next, we discuss the reason why the error in the proposed method is smaller than that in the comparison method. In the proposed method, even for the frames where the CNN is not executed, the hand position recognition at 1000Hz by the tracking process can output values close to the reference data. On the other hand, the comparison method outputs the last CNN estimation result without updating it for the frames where the CNN is not executed, and thus, the updating in the hand position recognition is limited to 100Hz. The effectiveness of the proposed method for fast hand tracking is the reason why the proposed method has superior accuracy.

### 4.2. Grasp Type Estimation

This subsection explains the experiment and evaluation for grasp type estimation based on the finger position recognition described above.

#### 4.2.1. Preparation for the Experiment

We trained a decision tree that outputs a grasp type label using the hand position as input. First, we captured 49 images of a power grasp, 63 images of a precision grasp, and 52 images of a non-grasp and annotated them with the grasp type label. Next, we annotated the hand positions in the images by estimating the CNN trained in the above subsection. After preprocessing, we transformed the hand positions represented in the Cartesian coordinate system into a polar coordinate system centered on the palm of the hand and normalized them by hand size to obtain 10 variables. The number of variables in the decision tree was two: length *r* and angle θ for each of the five types of fingers (index, middle, ring, pinky, and thumb). The length *r* is the ratio of the distance from the center of the palm to the tip of the middle finger with the fingers extended to the distance from the palm to each fingertip. The angle θ is the angle between the finger and the middle finger, with the thumb direction being positive and the little finger direction being negative.

Based on the above variables as inputs, a decision tree was trained using the leave-one-out method [37]. The model was trained with the depth of the decision tree from 1 to 10. As a result, when the depth was three, the accuracy was about 0.94, which can be considered to be sufficient. Thus, we decided that the depth of the tree structure should be set at three. Furthermore, the parameters of the decision tree such as the classification conditions, Gini coefficient, and depth were obtained.

#### 4.2.2. Experiment—1-B

We used a series of images of the grasping motion to evaluate the learned decision tree. We took a series of 500 frames of hand images of each type of grasping motion, starting from the open fingers, performing a power or precision grasp, and then, opening the fingers again. The exposure time of the high-speed camera was 0.5ms, the image size 400 pixels (width) × 300 pixels (height), and the frame rate 1000 fps. Based on the hand positions in the images recognized by the CNN and hand tracking (accurate mode), we performed the grasp type estimation using the decision tree and calculated the correct answer rate.

#### 4.2.3. Result

**Figure 10:**
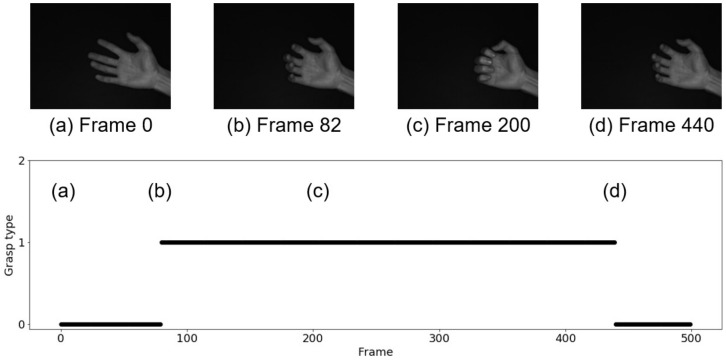
Estimation result in the case of power grasp.

**Figure 11:**
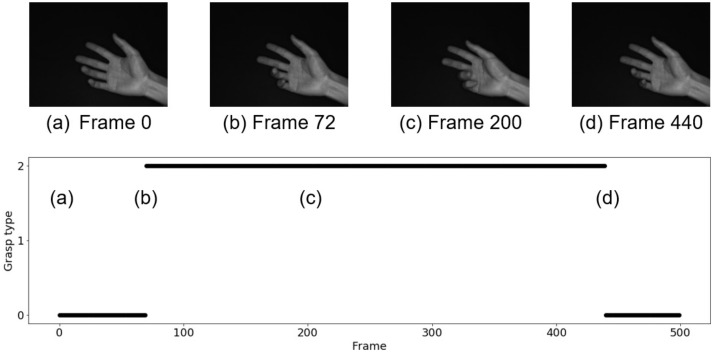
Estimation result in the case of precision grasp.

Figure 10 and Figure 11 show the results of grasp type estimation for a series of images and the hand image in a representative frame. The horizontal axes in Figure 10 and Figure 11 represent the frame number, and the vertical axis also represents the label of the grasp type, where 0 indicates non-grasp, 1 indicates a power grasp, and 2 indicates a precision grasp. Figure 10 is the result for the power grasp motion, which is a non-grasp at Frame 0 (a), judged as a power grasp at Frame 82 (b), a continued power grasp (c), and a non-grasp again at Frame 440 (d). Figure 11 is the result for the precision grasp motion, which is a non-grasp at Frame 0 (a), judged as a precision grasp at Frame 72 (b), a continued precision grasp (c), and a non-grasp again at Frame 440 (d). No misjudgments occurred in either the power grasp or the precision grasp experiments.

#### 4.2.4. Discussion

First, we discuss the stability of the grasp type estimation by the proposed method. From the results shown in Figure 10 and Figure 11, there was no misjudgment of the grasp type estimation. In addition, high-accuracy grasp type estimation was achieved successfully.

Next, we describe the processing speed of the grasp type estimation. The mean and standard deviation of the inference speed for three trials of 1000 consecutive inferences were 0.07ms and 0.02ms, respectively. This is much shorter than 1ms and is fast enough for a system operating at 1000Hz.

**Figure 12:**
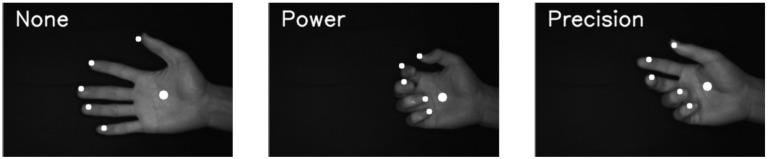
Difference among non-grasp, power grasp, and precision grasp and fingertip and palm positions (white circles).

Finally, the hand position recognition and grasp type estimation for the hand images evaluated in Experiment 1-A and 1-B are shown in Figure 12. From this result, we can conclude that the effectiveness of the proposed method for the hand position recognition and grasp type estimation is confirmed.

### 4.3. Teleoperated Grasp by Robot Hand

This subsection explains the experiment and evaluation for teleoperated grasping by a robot hand on the basis of human hand motion sensing.

#### 4.3.1. Experiment—2

In this experiment, the robot hand grasped a Styrofoam stick-shaped object with a diameter of 0.05 m and a length of 0.3 m, which was suspended by a thread in the robot’s range of motion. The human operator performed a “power grasp” or a “precision grasp” with his/her hand in the field of view of the high-speed camera. The real-time controller calculated the reference joint angles of the robot hand for the grasping operation according to the received grasp type and provided the reference joint angles as step inputs to the robot hand through the real-time control system.

#### 4.3.2. Results

**Figure 13:**
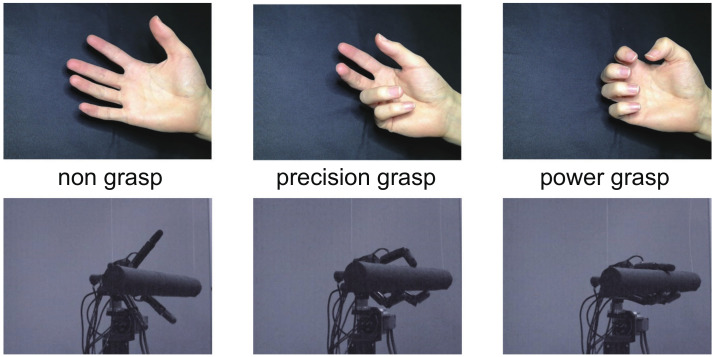
Experimental result of teleoperation; left, middle, and right show non-grasp, precision grasp, and power grasp, respectively.

A video of the grasping process of the robot hand can be seen at our web site [38], and the hand fingers of the robot hand and the operator are shown in Figure 13. From this result, we confirmed the effectiveness of the proposed teleoperation of the robot hand based on the hand tracking and grasp type estimation.

#### 4.3.3. Discussion

Since the operating frequency of both image processing and robot control was 1000Hz in the experiment, the operating frequency of the entire system was 1000Hz. Therefore, the robot hand manipulation with high-speed image processing proposed in this study achieved the target operating frequency of 1000 Hz.

Next, we consider the latency of the entire system. We define the latency as the time from the imaging to the completion of the robot hand motion. That is to say, the latency can be evaluated from the total time for the image acquisition, image processing, including hand tracking and grasp type estimation, transmitting the results from the image-processing PC to the real-time controller, and implementing robot hand motion.

**Figure 14:**
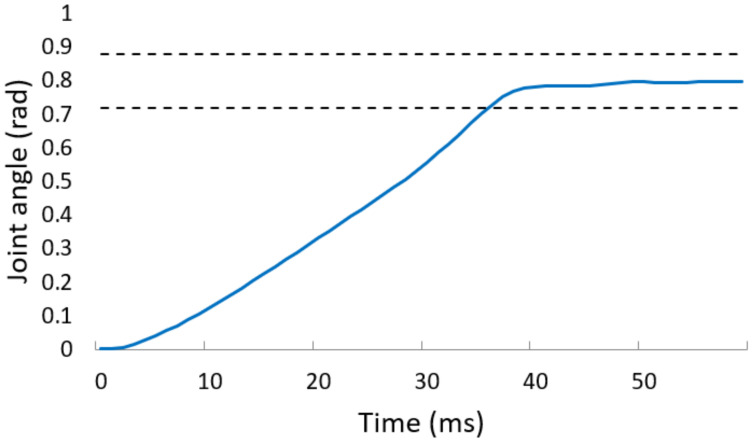
Joint response of high-speed robot hand.

First, the latency from the end of imaging to the end of transmission of the image processing result was 1 ms. Second, the latency from the transmission of the result by the image-processing PC to the output of the control input by the real-time controller was from 2∼3 ms, and the worst case was 3 ms. Next, we need to consider the latency from the output of the control input to the completion of the robot hand motion. The time required to converge to ±10% of the reference joint angle is shown in Table 3. From the top in Table 3, “Joint” means the root and tip links of the middle finger and the root, tip, and rotation of the left and right thumbs around the palm, respectively. Furthermore, Figure 14 shows the step response of the tip link of the left and right thumbs from an initial value of 0.0rad to a reference joint angle of 0.8rad. The dashed line depicts the range of ±10% of the reference joint angle 0.8rad, and the slowest convergence time was 36ms. Therefore, it took 36ms to complete the grasp after the real-time controller received the grasp type. As described above, the latency of the entire system was 1 ms for the imaging and the grasp type estimation, 3 ms for the communication between the image-processing PC and the real-time controller, and 36 ms for the robot operation, totaling 40 ms for the teleoperation of the robot hand. Since this value (40 ms) is close to the sampling time of the human eye (around 33 ms), our developed system is fast enough for robot teleoperation.

**Figure 15:**
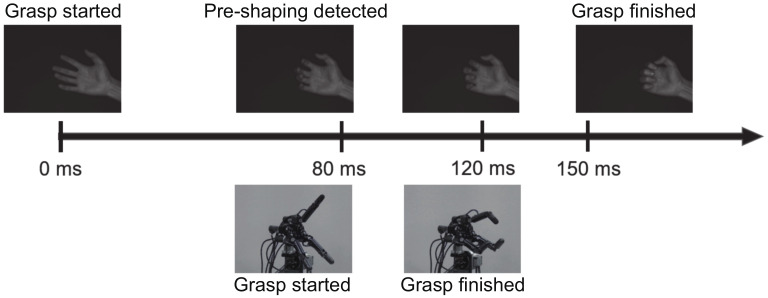
Time series of human hand motion and robot hand motion.

Figure 15 shows the timeline of the human and robot grasping motions. From the experimental results, it took about 80ms from the time the hand starts the grasping motion to the time the hand form that is estimated to be a specific grasping form is captured and 150ms until the time the motion is completed. On the other hand, it took 40ms for the robotic hand to complete the grasp after the hand configuration that is estimated to be a specific grasp configuration is captured. In other words, the grasping by the robotic hand is completed when the grasping motion by the fingers is completed at 80ms, and the remote grasping operation using the robotic hand is realized by anticipating the motion from the human hand morphology during the grasping motion, called pre-shaping. Consequently, we achieved teleoperated robot hand control beyond the speed of human hand motion by using the proposed method and system. This result may contribute to compensate the latency due to the network in the teleoperation.

## 5. Conclusions

The purpose of the work described in this paper was to develop an intuitive and fast telerobot grasping and manipulation system, which requires fast recognition of the intended grasping method from the operator’s gestures. In this paper, we proposed a method for fast recognition of grasping intentions by obtaining hand positions from gesture images and estimating the grasp type from the hand positions by machine learning. In particular, we combined machine learning and tracking to achieve both high speed and accuracy in hand position acquisition. In the evaluation experiments of the hand position recognition method, we achieved a mean-squared error of 1.06pixel, an operating frequency of 1000Hz, and a latency of 1ms. In the evaluation experiment of the grasp type estimation, we also achieved an accuracy of 94%, and the inference time was 0.07ms. These results show that the operating frequency of the system from gesture capturing to grasping form estimation was 1000Hz and the latency was 1ms, which confirms the effectiveness of the proposed method. As a result of the remote grasping operation experiment by the high-speed robot hand using the high-speed grasping form estimation system, the grasping operation was completed in 40ms after the hand image was captured. This is the time when the grasping operation by the fingers was completed at 80%, and the high-speed tele-grasping operation was realized successfully.

The first application of the system proposed in this study is HMI, which uses high-speed gesture recognition. In this study, the grasping form was obtained from the hand position. However, the proposed method can be applied to HMI that supports various hand gestures because it is easy to obtain other types of gestures. Next, human–machine coordination using fast and accurate hand position recognition can be considered as an application. Since the hand positions are acquired with high speed and high accuracy, the system can be applied to human–machine coordination using not only gestures, but also hand positions, and remote master–slave operation of robots by mapping.

There are still some issues to be solved in this research. One of them is the 3D measurement of the hand position. Currently, the hand position is only measured in two dimensions, which restricts the orientation of the operator’s hand, but we believe that three-dimensional measurement will become possible by using multiple cameras, color information, and depth information. The other is hand position recognition against a miscellaneous background. In this study, the background of the hand image was black, but by training the machine learning model using images with various backgrounds, the hand position can be recognized without being affected by the background, and the applicability of the system will be enhanced. These issues described above will be solved in the future.

## Figures and Tables

**Table 1:** Positioning of this research.

Evaluation Index	Conventional Method	Proposed Method
Comfortable operation	Contact	Non-contact
Application to various robots	Motion mapping	Intention extraction
Fine-motion recognition	Low-speed	High-speed

**Table 2:** Mean-Squared Error (MSE) of finger positions estimated by CNN w/ and w/o finger tracking.

Finger	MSE	
	with Finger Tracking/Pixel	without Finger Tracking/Pixel
Index	0.95	1.03
Middle	0.97	1.24
Ring	1.19	1.60
Pinky	1.18	1.47
Thumb	0.99	1.02
Average	1.06	1.27

**Table 3:** Time for convergence of robot hand joint angle to ±10% of the reference angle.

Finger	Joint	Time/ms
Middle finger	root	25
	top	25
	root	26
Left and right thumbs	top	36
	rotation around palm	18

## Data Availability

Not applicable.

## Appendix A

In this Appendix, we show an algorithm of another mode, which is a high-accuracy mode for finger tracking, shown in Algorithm A1.

**Algorithm A1** Finger tracking with high accuracy
1: framenumber←0 2: **while** True **do** 3: **if** framenumber(modT)≡0 **then** 4: **repeat** 5: wait 6: **until** resultCNN is received 7: calculate $C_{n}$ 8: resultFT←resultCNN 9: **else** 10: calculate $C_{n+k}$ 11: $resultFT\leftarrow resultCNN+C_{n+k}-C_{n}$ 12: **end if** 13: send resultFT 14: framenumber←framenumber+1 15: **end while**
